# Xanthophyll pigments dietary supplements administration and retinal health in the context of increasing life expectancy trend

**DOI:** 10.3389/fnut.2023.1226686

**Published:** 2023-08-10

**Authors:** Sanda Jurja, Ticuta Negreanu-Pirjol, Monica Vasile, Mihaela Mehedinti Hincu, Valeria Coviltir, Bogdan-Stefan Negreanu-Pirjol

**Affiliations:** ^1^Department of Ophthalmology, Faculty of Medicine, “Ovidius” University, Constanta, Romania; ^2^Department of Pharmaceutical Sciences, Faculty of Pharmacy, “Ovidius” University, Constanta, Romania; ^3^Department of Preclinical Sciences, Faculty of Medicine, “Ovidius” University, Constanta, Romania; ^4^Department of Histology, Faculty of Medicine, “Dunarea de Jos” University, Galati, Romania; ^5^Department of Ophthalmology, Faculty of Medicine, “Carol Davila” University of Medicine and Pharmacy, Bucharest, Romania

**Keywords:** xanthophyll pigments, dietary supplements, visual health, retina, degeneration

## Abstract

**Introduction:**

Medicine faces nowadays the trend of increasing life expectancy of human population, with the resulting increase of degenerative age related diseases prevalence, combined with the risks of less tempered sun radiations environment exposure. Under these circumstances, our work pointed out on evaluating the effect of some xanthophyll pigments dietary supplements, actually widely recommended, for prevention of retinal degenerative damages and for slowing down the progression of such age related changes if they have already occurred. These dietary supplements are already well known for their total antioxidant activity, proven by photochemiluminescence method using Total Antioxidant Capacity in Lipid soluble-substances procedure.

**Materials and methods:**

The study recruited a number of 120 subjects equally divided on genders. The lot included a first group of 60 patients with comparable ages (all of them over 50 years and divided in 2 segments of age: 50-60 and over 60) and suffering from comparable retinal age-related degenerative abnormalities (mild/medium severity age-related macular degeneration according to Wisconsin Age-Related Maculopathy Grading System), and a second group, considered control, including a similar number of healthy, normal retina subjects belonging to same age and gender categories. There were evaluated at baseline the eye medical status and the retinal risk by specific methods: complete eye check-up, Amsler grid, specific standardized questionnaires focused on visual function and its impact on the quality of current life. Both groups, patients and control, received similar dosages of xanthophyll pigments dietary supplements including lutein and zeaxanthin during 18 months after baseline; at the end of this supplementation period a new evaluation was conducted. In the second part of the research all subjects involved received a new dietary supplement in which the same xanthophylls were enriched with C and E vitamins and oligo-elements Zinc and Copper. At the end of three years duration supplementation, the subjects were reevaluated and the paper presents the conclusions on the matter, pointing on the impact of xanthophyll supplements on visual health.

**Results:**

Correlation tests were applied to the complete set of data. Correlation tests have values between -1 and +1. The value -1 represents the negative correlation (reverse proportionality) meanwhile the value +1 represents the positive correlation (direct proportionality). The charts show the curves that are fitting experimental data. The dependence is linear in nature, and the value R2, as it approaches more the value 1, represents a better match with the experimental data (the data are in a percentage of approximately 99% on these straight lines of type y = ax + b). In the charts, there were noted the average values of the scores for healthy control patients with “Control”, and the average values of the scores for the patients with existing age related degenerative retinal pathology at baseline with “Patients”.

**Discussion:**

The retinal function and the impact of visual condition on health were both evaluated at baseline, 18 months and 36 months after baseline, by visual acuity, ophthalmoscopy fundus examination, Amsler test and by asking the subjects to answer the visual function questionnaires: EQ-5D, NEI-VFQ-25, as measures of health status quality and of the influence on welfare. The study revealed that under supplementation both control healthy subjects and patients with known degenerative retinal pathology included in the 50-60 years of age group evolved almost the same way, leading to the conclusion that administered xanthophyll pigments-based supplements, simple or enriched, managed to slow down the progression of abnormal degenerative vision loss to a rate comparable to physiological aging-related vision loss. It was also observed that intake of xanthophyll pigments dietary supplements preserved the general health condition and maintained relatively constant vision on the entire 36th months follow-up research duration in patients presented with existing age related degenerative retinal pathology at baseline. For healthy subjects, evaluation showed an improvement in results after dietary supplementation, with maintenance of constant vision and a significantly increase of general condition, in a positive sense. For subjects over the age of 60 dietary supplements intake was even more effective compared to younger group in providing better control of degenerative processes.

## 1. Introduction

The role of food in preserving ocular health was mainly ignored for many years except for the known classical recommendation of consuming carrots. The focus of researchers on detecting valuable correlations of nutrition, dietary supplements intake and ocular health developed quite recently, with encouraging results despite the small volume of definitive data. Antioxidants, carotenoids or various nutrients might have a good impact on pathology related to aging processes of the ocular tissues, ranging from macular degeneration to dry eye syndrome (1).

Though it would be obviously premature to develop distinctive guidelines on dietary supplements targeted at those worried about the quality and preservation of visual function, what is effective in prophylaxis of cardiovascular disease and cancer might also work in combating visual deterioration, as the identical mechanism of oxidative stress, common in the pathogenesis of cardiac pathology and various age-related conditions, seems to alter ocular health. The mechanisms of occurence of many ocular ageing-related phenomena are as yet incompletely elucidated, however there is nowadays increasing acceptance of the impact of certain factors in the development of these phenomena: oxidative aggression, inflammation, toxicity from blue light cumulated exposure, retinal pigment epithelium (RPE) cells malfunction, poor blood irrigation in the foveal choroid (1).

The idea that oxygen, the vital need for all living organisms, is equally associated with a toxic potential gets more and more widely clarified and agreed. There is increasing data supporting the destructive influence of reactive oxygen intermediates (ROI) in pathologies related to ageing of ocular tissues, the notion of oxidative stress including all damage caused by unstable and reactive oxygen metabolites (2).

Oxidative stress occurs when imbalances occur in the body between the production of reactive oxygen (including free radicals) and the detoxification capacity of reactive intermediates. Basically, when a cell is exposed to more reactive oxygen compounds, it can degrade.

Thus, oxidative stress contributes to ageing processes and the pathophysiology of degenerative diseases.

Depending on their chemistry, their origin location, their tropism onto certain targets in the body, their allegiance to the free radical or non-radical subgroups, ROI can be categorised. There are many types of free radicals. In humans, the most significant are free oxygen radicals (reactive oxygen species). Examples include singlet oxygen, hydrogen peroxide, superoxides and hydroxyl anions. There are two common forms of free radicals: reactive oxygen species (ROS) and reactive nitrogen species (RNS). Examples of ROS include: superoxide anion, hydrogen peroxide, highly reactive hydroxyl radical and peroxyl radical. RNS are often considered a subclass of ROS and include: nitric oxide, nitrous oxide, peroxynitrite, nitroxyl anion and peroxynitrous acid.

Free radicals are atoms or molecules that contain odd electrons, which tend to reach chemical stability. The process can involve a number of reactions. When a free radical ‘steals’ an electron from a molecule, that molecule becomes a free radical because it is missing an electron - and so on, generating a veritable cascade of cytotoxic reactions. Free radicals can damage the body’s DNA, which contains genes as well as proteins, lipids, cell membranes, causing disease. Antioxidants help to maintain physiological levels of free radicals to maintain their physiological function and prevent pathological effects caused by the action of oxidative stress, as this is precisely the state of imbalance between ROS and the properties of antioxidants. In these circumstances, ROS outperform antioxidants due to increased levels, deficient antioxidant defence or a combination of the two, attacking biological structures (3).

The retina is particularly exposed to altering by ROI aggression (4, 5). This particular sensitivity is due to several factors. A first such factor is the very high oxygen consumption in the retina. In addition to this, there is the massive presence of polyunsaturated fatty acids (PUFA) in the photoreceptor structure of the retina. The chemical structure of PUFA includes hydrogen atoms, which provide an electron, the perfect target for ROI and thus for oxidative destruction. In addition, by definition, retinal function involves photosensitization phenomena under the action of visible radiation, which is certainly ROI-producing. The phagocytosis function of the RPE also is the one that generates hydrogen peroxide (non-radical species).

Lutein and its stereoisomer, zeaxanthin, belong to the carotenoids xanthophyll group.

Compared to hydrocarbon carotenoids, for example β-carotene and lycopene, lutein and zeaxanthin have two hydroxyl groups, on both sides of the molecule. Both groups have an essential contribution regarding their biological role and in identifying appropriate chemical methods for the determination of these xanthophylls.

Xanthophylls such as Lutein (3R, 3′R, 6′R)-β,ε-carotene-3,3′-diol), MW: 568.88 g/mol and Zeaxanthin all-trans-(3R, 3′R)-β-carotene-3,3′-diol), MW: 568.9 g/mol, with identical molecular formulas (C40H56O2), isomers, but not stereoisomers, distinguished strictly by location of the double bond in one final ring, emphasize a specificity given by their presence as carotenoids in certain eye tissues, being strongly represented in the macula, a little portion of the retina in charge with central vision and visual accuracy. Lutein and zeaxanthin exist in the lens, another eye tissue essential for vision (6). Currently, macular degeneration is the leading factor for vision loss in populations from developed regions, defined as progressing, degenerative, non-reversible damages of the central retinal zone (macula), which is responsible for detailed vision. It has a yellowish coloration given by a yellow pigment. Macular degeneration develops progressively affecting over 5% of people over 65 years of age, so that it tends to become a public health problem of the 21st century.

The dry form of macular degeneration has no effective treatment yet but is responsive to nutraceuticals including vitamins and minerals or lutein and zeaxanthin, as major carotenoids concentrated in the macula of human retina (6). Lutein and zeaxanthin are known as plants generated pigments, found in yellow to reddish colour fruits and vegetables. Both are chemically pretty alike, very slightly differentiated by the atomic layout of the molecules. Each are strong antioxidants and provide plenty of positive impacts on health condition, specially known for eyes protection (6, 7).

Lutein is present in many biological systems, such as bacteria, algae, yeasts, plants, usually existing in flowers, grains, fruits and vegetables (8, 9). Meanwhile lutein is present in foods of animal and fish origin, and as a pharmacy nutritional supplement, so that we can refer to a multiple choice lutein market (10). Carotenoids are very healthy due to their high antioxidant activity (11). Lutein and zeaxanthin prevent and limit the ocular damage from UV radiation and contribute decisively for brain development (12–14). Other carotenoids have the capacity to prevent the appearance of low-density lipoprotein (LDL) and thus contribute towards heart protection (15–17).

### 1.1. Oxidative stress has major impact in the age-related macular degeneration

Pathogenesis (18, 19). Some studies emphasize the antioxidative capacity of astaxanthin, zeaxanthin, lutein, along with ascorbic acid and tocopherol acetate, by various procedures including spectrophotometric, fluorimetric and chemiluminescence methods, confirming that xanthophylls have an increased antioxidant potential. Due to this property, they can be assigned the first option position in combating retinal oxidative damage, an important step in preventing or slowing down the progression of AMD (20).

Zeaxanthin, a non-provitamin A carotenoid similar to lutein, was demonstrated to have significant positive impact on human health due to its capacity to capture and neutralize free radicals, providing antioxidant effects and reducing inflammation. This carotenoid presents beneficial impact on eye, skin, liver and cardiovascular health (21).

Literature emphasizes that lutein and zeaxanthin have a special affinity for RPE (retinal pigment epithelium) cells (22). Moreover, it was found that ARPE-2 cells (a human RPE cell line), same as RPE cells had a double affinity for lutein and zeaxanthin compared to the affinity for beta-carotene, when treated with 3 different pigments (23).

Macula is a specialized region in the retina of humans, centered by the foveola, which provides the clearest vision. Due to their major presence inside macular retina, lutein and zeaxanthin provide a protector shield against blue light (24). They behave as powerful antioxidants and neutralize ROS resulting from photoexcitation (25, 26). Degeneration processes can damage macula, mostly in subjects over 65 years of age. This risk highlights the importance and necessity to increase the dietary intake of lutein as a strategy to reduce the incidence of macular degeneration and cataracts (25, 27–29).

The carotenoid profile of human cells is known to include six such pigments; to those already mentioned above are added α-carotene, β-carotene, β-cryptoxanthin and lycopene (30, 31). Human plasma includes several such pigments, depending on diet, while few pigments can concentrate in certain tissues, as is the case with lutein and zeaxanthin in macula lutea (32–36).

The possibilities to identify, quantify and monitor the antioxidative activity of various molecules *in vivo* are reduced due to the unavailability of suitable biomarkers. For the moment investigators have no method to evaluate oxidative stress reaction and total antioxidant activity in the animal kingdom. All that can be detected and measured so far are lipoprotein fragments from animals or humans that have consumed carotenoids through food or supplementation. This method has been used by researchers quite recently and involves introducing carotenoids into the LDL molecule or target membrane. It has been found that increasing carotenoid intake through increased fruit and vegetable intake or supplementation decreased the degree of oxidation of LDL particles (37–39).

However, it should be noted that higher consumption of fruit and vegetables increases plasma levels not only of carotenoids, but also of vitamin C, polyphenols and flavonoids, which are also agents with antioxidative activity and to which any decrease in LDL oxidation can be attributed (40). Other research has demonstrated the presence of antioxidant activity also in the case of lycopene and other carotenoids (41), with the caveat that some authors found that the association of lutein or lycopene to beta-carotene, with already demonstrated antioxidant efficacy, paradoxically resulted in an amplification of LDL oxidation (42).

Consequently, carotenoids known as tetraterpenoids behaved as a shield to protect photosynthetic structures against oxidative stress induced by ROS (43). Their functions in nature are multiple, involving growth, signaling oxidative stress, determining sex-linked color patterns or constituting a precursor of vitamin A for numerous vegetal varieties (44–46). It is precisely because of these defensive capacities that carotenoids have led to the idea of a correlation between their concentrations in the body and the prophylaxis or therapy of many types of pathology (47–50).

Other studies revealed that lutein has higher antioxidative activity than different carotenoids, both *in vivo* and *in vitro*, being able to neutralize superoxide and hydroxyl radicals, and to block lipid peroxidation (51).

Lutein also significantly decreased the destructive impact of oxidative stress by reducing membrane permeability for oxygen (52). Other research has demonstrated the superior antioxidant efficacy of lutein compared to β-carotene in combating auto oxidation of lipids in cell cultures (53). Lee and collaborators have shown the anti-inflammatory and immunosuppressive effect of dietary lutein (54, 55). Studies have also shown the antioxidant defensive action of lutein on liver cells in humans (56, 57).

Starting from scientific literature regarding a few studies on total antioxidant capacity through photo chemiluminescence method, applied of two type antiaging vegetal dietary supplements such as Lutein, Zeaxanthin (58–66) the goal of this study was to highlight variations of treatment response potentially connected to different antioxidative potential of concerned antiaging vegetal dietary supplements, consisting in soft capsules based on vegetal pigments (Lutein 10 mg + Zeaxanthin 2 mg), or same active principles combined with vasoprotective and antioxidant capacity components (vitamin C, vitamin E, Zinc and Copper) (67–71).

## 2. Materials and methods

In this study the impact of xanthophyll pigments dietary supplements administration has been studied by evaluating the retina, both morphological and functional.

Two groups of subjects were studied.

The first group included 60 subjects with mild/medium severity macular retina abnormalities - early age-related macular degeneration (AMD) according to Wisconsin Age-Related Maculopathy Grading System (AREDS 11- step severity scale), including 26 subjects (13 women +13 men) of 50–60 years of age and 34 subjects (19 women +15 men) older than 60 years.

The second group (control group) consisted of 60 subjects with healthy eyes: 30 subjects (15 women +15 men) of 50–60 years of age and 30 subjects (15 women +15 men) older than 60 years.

Both groups of subjects received xanthophyll pigments dietary supplements containing 10 mg of Lutein +2 mg of Zeaxanthin, for 18 months after baseline. Afterwards, the retinal function and the impact of visual condition on health were evaluated by asking the subjects to answer the visual function questionnaires.

In the second part of the study, the same groups of subjects received a different xanthophyll pigments dietary supplement, containing the same active principles as the first one, enriched with vitamin C, vitamin E, Zinc and Copper, for another 18 months duration.

At the end of this second supplementation period, a new evaluation was conducted.

The retina, as first peripheral nervous structure which provides visual sensation, was evaluated by visual acuity, visual field, Amsler Test, fundus camera images and macular pigment optical densitometry (MPOD apparatus). In order to quantify the impact of administered xanthophyll pigments dietary supplements on preservation of retinal abilities, subjects were asked to answer The EuroQol EQ-5D-5L Questionnaire. They were also asked to score on The EuroQol Visual Analogue Scale EQ-VAS Questionnaire. The 25 Items Visual Function Questionnaire VFQ- 25 was also applied to all involved subjects. All these questionnaires offer preference-based measure of health status which is frequently used in clinical trials, observational studies and other health surveys. They are standardized measures of health status, here including also the visual function, developed in order to provide a simple, generic measure of health for clinical and economic appraisal (72).

Resulting scores were afterwards collected, summarized and statistical analysis was carried out.

## 3. Results

Correlation tests were conducted on the complete set of data. Correlation tests have values between −1 and + 1. The value −1 represents the negative correlation (reverse proportionality) meanwhile the value +1 represents the positive correlation (direct proportionality). The charts show the curves that are fitting the experimental data. The type of dependence is linear in nature, and the value *R*^2^, as it approaches more the value 1, represents a better match with the experimental data (practically shows that the evolution of the data is of a linear type, and the data are in a percentage of approximately 99% on these straight lines of type y = ax + b).

In the following charts, there were noted the average values of the scores for healthy subjects with “control,” and the average values of the scores for the patients with existing age related degenerative retinal pathology at the time of presentation to the ophthalmologist, with “patients.”

**Figure 1:**
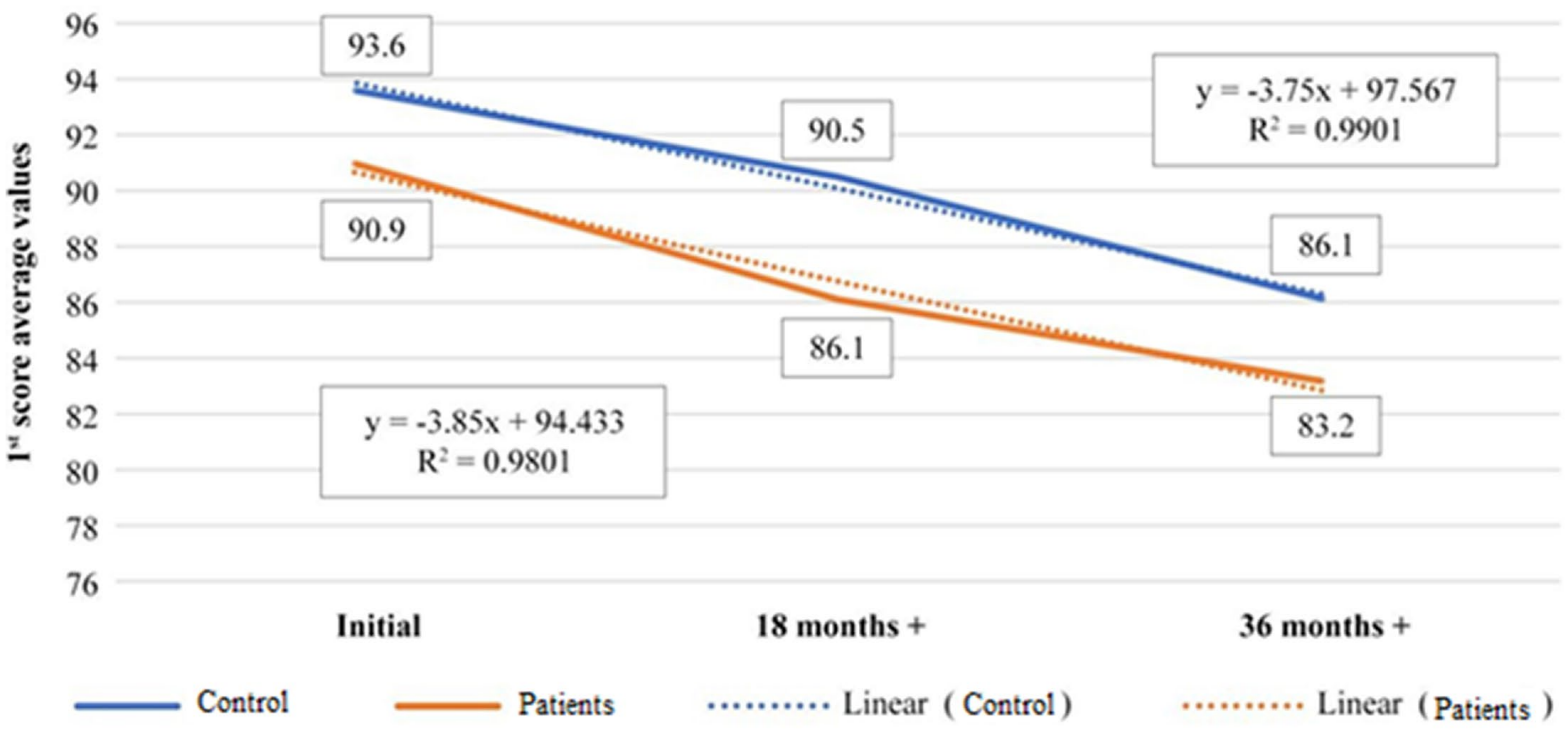
Representation of evolution of 1st score average value, during 36th months treatment time for 50–60 years old age category, control versus patients.

The first chart represents the evolution of 1st score for the 50–60 years age category, starting from the day of the first eye check-up until the end of the 36th months xanthophyll pigments-based dietary supplementation. The value *R*^2^ shows a match of the experimental data with the fitting curve in proportion of 99% for the control and 98% for the patients, and the value of ‘b’ from the equation y = ax + b, represents the slope of the fitting curve. This means, the evolution of the data is almost similar, the slope difference of the linear fitting curve being very small, b = 97.567 for control and b = 94.433 for the studied patients (Figure 1; Table 1).

**Table 1:** Average values of 1st score for 50–60 years old age category, control versus patients.

50–60 years old		Initial	18 months+	36 months+
	Control	93.6	90.5	86.1
	Patients	90.9	86.1	83.2

The correlation between the patients and controls in the age group 50–60 years is strongly positive (0.97; Table 2), which means that the two curves are correlated, meaning that both healthy subjects and those with known pathology evolve in the same way as a result of the administered dietary supplementation.

**Table 2:** Positive correlation between control and studied patients (50–60 years old, age category).

		Patients
Control	1	
Patients	0.971039	1

The second chart refers to the relationship between the 1st score and the 2nd score for the same age group 50–60 years old. The data correspond to a linear evolution of the 1st score in proportion of 99%, while for the 2nd score the match with the linear evolution is 85%. The correlation test run for the two parameters for the controls, category 50–60 years old, shows a high negative correlation of the two scores for them which means that 1st score and 2nd score are in reverse proportionality relation.

**Figure 2:**
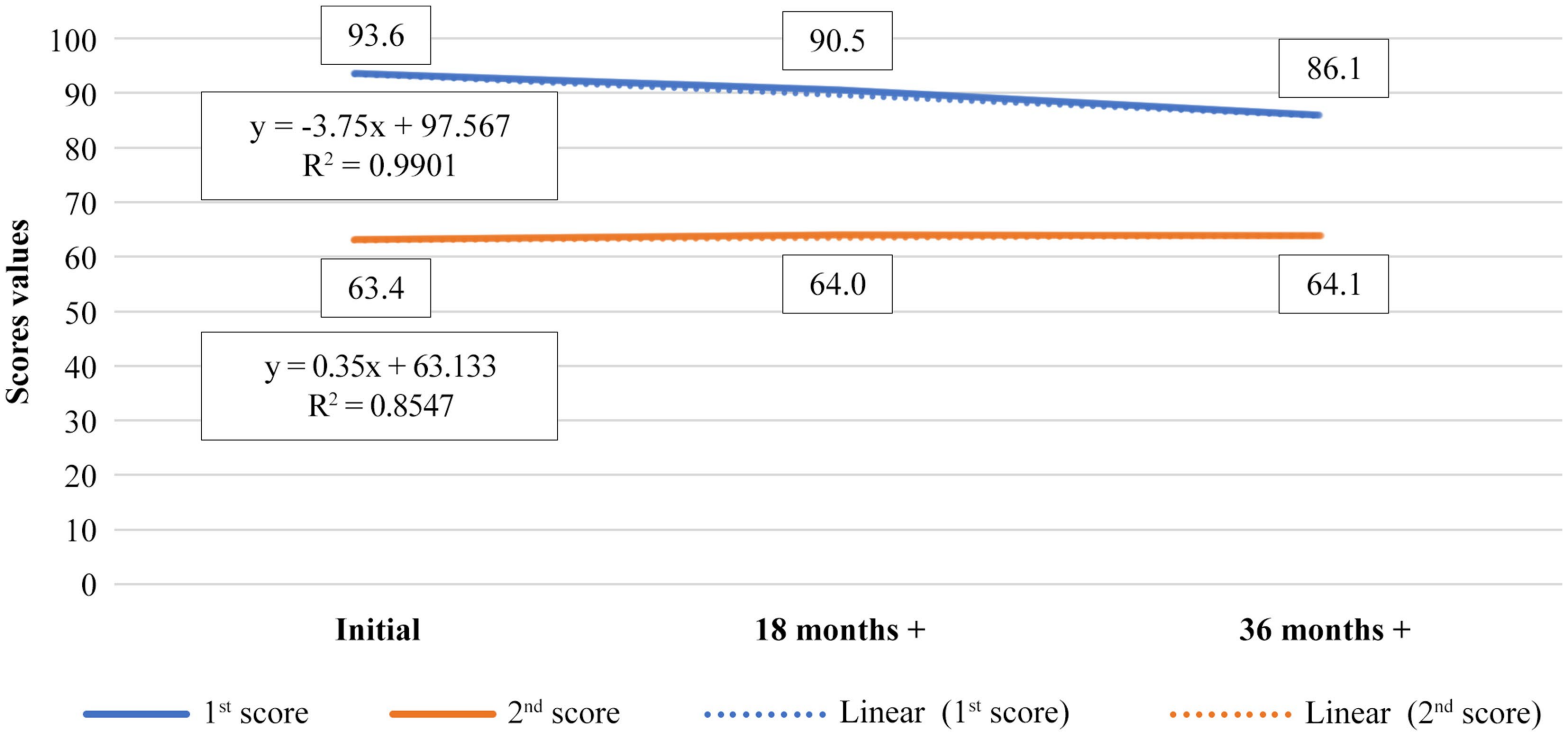
Representation of evolution of 1st score average value versus 2nd score average value, during 36th months treatment time for control, 50–60 years old age category.

The correlation coefficient is (−0.88; Table 3) while the maximum negative value can be (−1; Figure 2; Table 4).

**Table 3:** Negative correlation between control 1st and 2nd score (50–60 years old, age category).

	1st Score	2nd Score
1st Score	1	
2nd Score	−0.88192	1

**Table 4:** Average values of 1st and 2nd score for 50–60 years old age category, control.

Control 50–60 years old		1st Score	2nd Score
	Initial	93.6	63.4
	18 months+	90.5	64
	36 months+	86.1	64.1

It was observed that the general health condition associated with vision remains relatively constant for patients presented to the doctor with present pathology, while for healthy subjects (controls), 2nd score shows an improvement in results after supplementation, with a significantly increase of general condition, in a positive sense.

**Figure 3:**
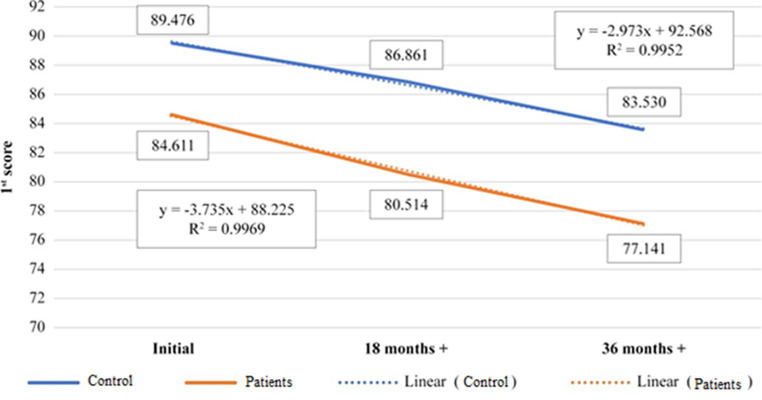
Representation of evolution of 1st score average value, control *vs* patients, during 36th months treatment time, 50–60+ years old age category.

Figure 3 refers to the entire set of data, controls and patients, both categories of age, 50–60 and 60+ years old. The average of 1st score over the 36th months dietary supplementation time, are fitting the linear evolution in percentage of 99% in both controls and patients. The slope of the fitted curve is slightly higher for the patients (b = 88.22) than for controls (b = 92.56). That means that the dietary supplementation as a whole is less efficient in patients with known pathology than in healthy subjects, but the difference is not significant, as the average t-test shows. The correlation coefficient is 0.99 (Table 5), meaning the data are in a strong positive correlation, both, controls and patients evolve similarly under dietary supplementation (Figure 3; Table 6).

**Table 5:** Positive correlation between control and patients 1st score (50–60+ years old).

50–60+ years old	Control	Patients
Control	1	
Patients	0.992159	1

**Table 6:** Average values of 1st score for 50–60+ years old age category, control vs. patients.

50–60+ years old		Control	Patients
	Initial	89.476	84.611
	18 months+	86.861	80.514
	36 months+	83.53	77.141

**Figure 4:**
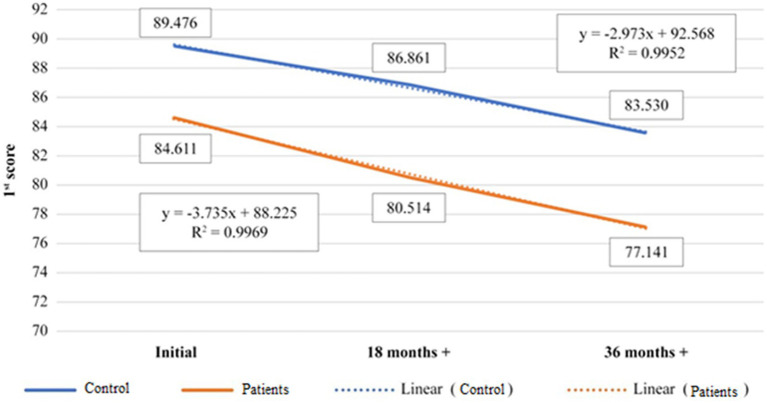
Representation of evolution of 1st score average value, control versus patients, during 36th months treatment time, 60+ years old age category.

Figure 4 refers to the evolution of the 1st score for the age category 60+ years old. The comparison made between controls and patients average values of the 1st score over the entire dietary supplementation period, is fitting the linear evolution in proportion of 99%, for both categories. The slope of the fitting curves for the patients is higher (b = 89.967) than in controls (b = 92.867), but the data are positively correlated to 99% (Table 7), meaning that both sets of data analyzed are in direct relation and the results are closely dependent (Figure 4; Table 8).

**Table 7:** Positive correlation between control and patients 1st score (60+ years old).

	Control	Patients
Control	1	
Patients	0.999996	1

**Table 8:** Average values of 1st score for 60+ years old age category, control *vs* patients.

Category 60+ years old		Control	Patients
	Initial	89.5	85.8
	18 months+	87.3	81.9
	36 months+	84.85	77.6

**Figure 5:**
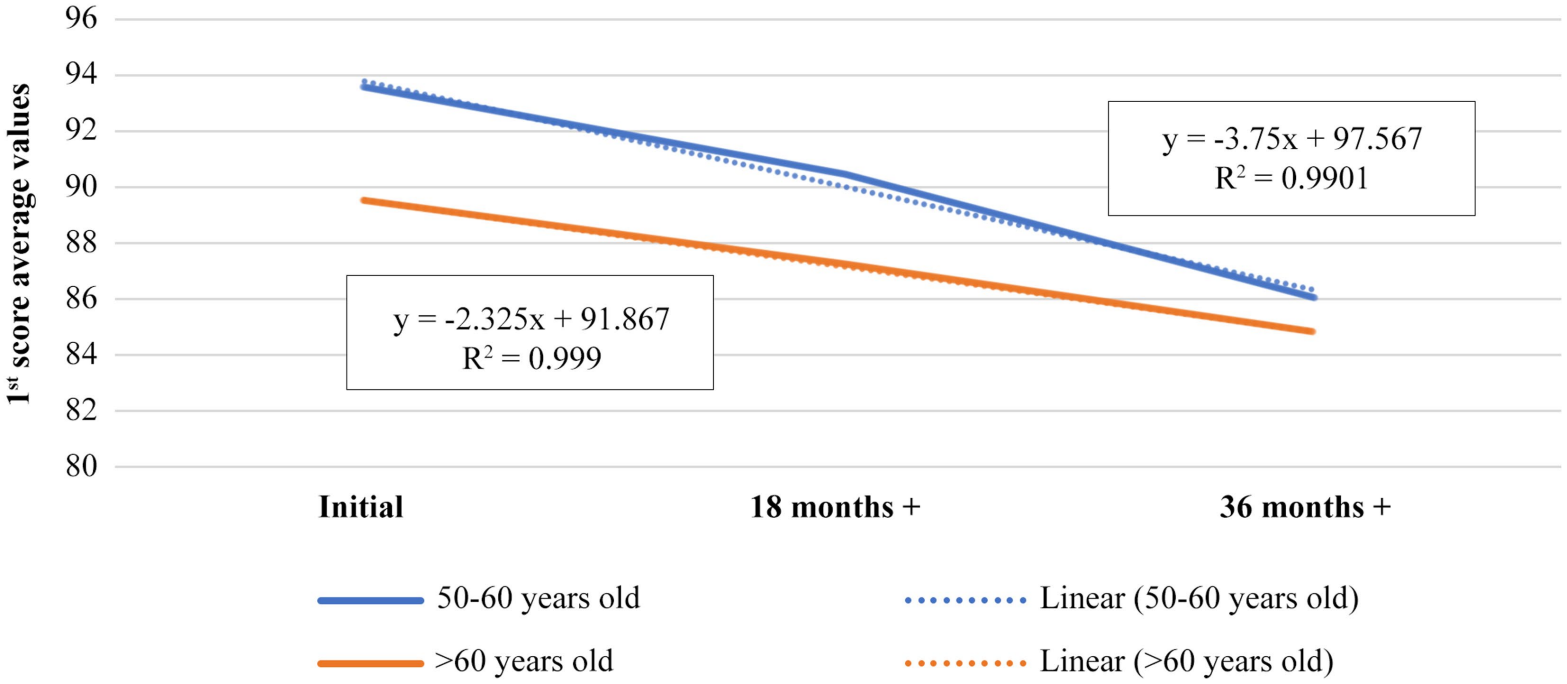
Representation of evolution of 1st score average value for patients, during 36th months treatment time, 50–60 versus 60+ years old age category.

Figure 5 shows linear dependence of 1st score with a match of 99%, but the slope of the 60+.

age category curve is lower (b = 91.867) than 50–60 age category (b = 97.567). This means that dietary supplementation is more effective in the case of 60+ age category, or that degenerative processes slow down with age (presumably due to age slowing down local metabolism), the dietary supplementation having better result in subjects over 60 years old. The correlation of the results of 1st score for the two age categories is also highly positive (0.997; Table 9), meaning that there is a direct proportionality between them and the evolution is positive for both, but the results are better for 60+ age category (Figure 5; Table 10).

**Table 9:** Positive correlation between the values of the patients 1st score, 50–60 versus 60+ years old category.

1st score	50–60 years old	60+ years old
Patients 50–60 years old	1	
Patients > 60 years old	0.99764	1

**Table 10:** Average values of 1st score for patients, 50–60 vs. 60+ years old age category.

1st Score	Patients	Initial	18 months+	36 months+
Patients	50–60	93.6	90.5	86.1
Patients	>60	89.5	87.3	84.85

Score 2 for the patients (subjects with existing degenerative retinal pathology) evolves upwards (in a negative sense) for both age categories, with a faster evolution in the age period 50–60 years. After 60 years the rate of deterioration of health in relationship with sight, is somewhat slower. Score 3 has an almost identical evolution in both age categories, the deterioration of near vision being maintained in approximately equal rates in patients.

Score 1 starts from significant differences between the patients and controls and evolves in the sense of health deterioration for both categories (with present pathology and those without initial pathology), with a slightly lower rate for controls than those with initial pathology.

The differences between the groups remain significant even after 36 months dietary supplementation, the degradation is higher in those who initially presented retinal degenerative pathology, and the data are in a positive correlation.

Score 2 shows an increasing evolution in the sense of decreasing health and vision with age, more pronounced at patients, while the controls 2nd score remains relatively constant throughout the supplementation, proving that xanthophyll pigments dietary supplementation manages to maintain the initial retinal state of healthy subjects throughout the study.

Score 3 shows a relatively constant evolution for the controls, which proves efficacy of xanthophyll dietary supplementation, and an increase in the patients, the growth rate being relatively small, although the difference between the initial and final state is significant.

## 4. Discussion

The retinal function and the impact of visual condition on health were both evaluated at baseline, 18 months and 36 months after baseline, by visual acuity, ophthalmoscopy fundus examination, Amsler test and by asking the subjects to answer the visual function questionnaires: EQ-5D, NEI-VFQ-25, as measures of health status quality, and of the influence on welfare.

The applied xanthophyll pigments-based dietary supplementation proves to be effective in case of people with existing degenerative retinal pathology, managing to keep a linear evolution of their visual health condition, the speed of progressive deterioration being slowed down. Under supplementation the vision decrease was done gradually with reduced speed, near to the age-related physiological rate.

It was no possible to compare the visual status evolution between subjects which received xanthophyll pigments dietary supplements and subjects who did not, because the authors of the research considered it unethical to deprive any study participant of a potentially beneficial and side effects-free supplement. However, the evolution of the 1st score, with strongly positive correlation and almost similar slope of the fitting curves for both patients and controls in the age group of 50–60 years showed that both healthy subjects and patients with known degenerative retinal pathology evolved almost the same way as a result of the administered xanthophyll pigments-based, simple or enriched, dietary supplements. This revelation of our data analysis brings a strong argument in favor of considering xanthophyll pigments dietary supplements a credible and necessary resource in the management of age degenerative retinal damages.

It was also observed, according to the 2nd score, that intake of xanthophyll pigments dietary supplements (both simple and enriched) preserved the general health condition and maintained relatively constant vision on the entire 36th months research duration for the patients presented to the doctor with existing age related degenerative retinal pathology at baseline. For healthy subjects, 2nd score showed an improvement in results after dietary supplementation, with a significantly increase of general condition, in a positive sense.

According to the average t-test, despite the fact that xanthophyll pigments dietary supplements as a whole are less efficient in patients with known retinal pathology than in healthy subjects, the difference is not significant, observation which leads us once more to the idea of a real positive effect.

Subjects who initially did not show retinal degenerative changes maintained their health and constant vision during xanthophyll pigment dietary supplements intake duration.

The studied dietary supplementation had better results for people over the age of 60, where the degenerative processes were better controlled under xanthophyll pigments intake, compared to younger groups. An explanation of this finding might be that retinal degenerative processes slow down with age (presumably due to age slowing down local metabolism). All the more this should encourage the administration of dietary supplements.

## Data availability statement

The original contributions presented in the study are included in the article/Supplementary material, further inquiries can be directed to the corresponding authors.

## Ethics statement

The studies involving human participants were reviewed and approved by Institutional Review Board Ethics Committee of Ovidius University Constanta. The patients/participants provided their written informed consent to participate in this study.

## Author contributions

SJ, TN-P, MMH, and B-SN-P were involved in literature research and wrote the manuscript. MV supported the statistical analysis and reviewed the results. SJ, TN-P, and B-SN-P conceived, planned, and followed the execution of the experiments. SJ and VC provided patient samples. All authors contributed to the article and approved the submitted version.

## Conflict of interest

The authors declare that the research was conducted in the absence of any commercial or financial relationships that could be construed as a potential conflict of interest.

## Publisher’s note

All claims expressed in this article are solely those of the authors and do not necessarily represent those of their affiliated organizations, or those of the publisher, the editors and the reviewers. Any product that may be evaluated in this article, or claim that may be made by its manufacturer, is not guaranteed or endorsed by the publisher.
