# Supplementary material for: Xanthophyll pigments dietary supplements administration and retinal health in the context of increasing life expectancy trend
Source: Front Nutr. 2023 Aug 10;10:1226686. doi: 10.3389/fnut.2023.1226686 (PMC10450221; doi:10.3389/fnut.2023.1226686)
Supplement: Supplementary file 1 [file Table_11.docx]

| PATIENTS | 60 AMD SUBJECTS | 50-60 YEARS | | > 60 YEARS | |
| --- | --- | --- | --- | --- | --- |
|  |  | 13 **♂** | 13 ♀ | 19 **♂** | 15♀ |
| CONTROLS | 60 HEALTHY SUBJECTS | 50-60 YEARS | | > 60 YEARS | |
|  |  | 15 **♂** | 15 ♀ | 15 **♂** | 15 ♀ |

STUDY DESIGN

Supplementation 1 – Lutein 10mg + zeaxanthin 2mg;

| Baseline Evaluation | I Evaluation | II Evaluation |
| --- | --- | --- |
| 0 | 18 moths | 36 months |

Supplementation 2 - Lutein 10mg + zeaxanthin 2mg + Vit.C, Vit.E, zinc, copper.
